# Immunological detection of human and camel cystic echinococcosis using different antigens of hydatid cyst fluid, protoscoleces, and germinal layers

**DOI:** 10.14202/vetworld.2021.270-275

**Published:** 2021-01-29

**Authors:** Mohey A. Hassanain, Nagwa I. Toaleb, Raafat M. Shaapan, Nawal A. Hassanain, Ahmed Maher, Ahmed B. Yousif

**Affiliations:** 1 Department of Zoonotic Diseases, National Research Centre, El-Tahrir Street, Dokki, Giza, Egypt; 2 Department of Parasitology and Animal Diseases, National Research Center, El-Tahrir Street, Dokki, Giza, Egypt; 3 Faculty of Medicine, Fayoum University, El Fayoum, Egypt

**Keywords:** cystic echinococcosis, Human, Camel, Hydatid cyst antigens, ELISA, SDS-PAGE, Western blot

## Abstract

**Background and Aim:**

Cystic echinococcosis (CE)/hydatidosis is one of the most prevalent neglected zoonotic diseases. It is initially asymptomatic and does not produce any clinical signs until the cyst becomes enlarged, causing localized pressure on internal organs and tissues. Therefore, the detection of *Echinococcus granulosus* antibodies is highly essential. This study evaluated the antigens of hydatid cyst fluid, protoscoleces, and germinal layers for efficient immunological diagnosis of CE in humans and camels.

**Materials and Methods:**

Hydatid cyst fluid (FLc), protoscoleces (Psc), and the germinal layer (GLc) antigens were prepared from camel-lung hydatid cysts. In the same way, hydatid cyst fluid (FLh) and protoscoleces (Psh) antigens from human-liver cyst aspirate were produced. The comparative immunodiagnostic efficacy of the prepared antigens was verified using indirect enzyme-linked immunosorbent assay (ELISA), SDS-PAGE, and immunoblotting.

**Results:**

ELISA proves that FLc and GLc antigens were higher than FLh and Psh antigens. This shows that binding reactivity in naturally infected human sera, camel sera, and Psc is the most potent, exhibiting 100% sensitivity with 78.26% and 76.47% specificity in camel and human sera, respectively. The CE prevalence using diagnostic Psc was 54.79% and 61.32% in tested human and camel sera, respectively. The electrophoretic profiles of all shared antigens showed similarities at 52, 41, and 22 kDa. Immunoblotting demonstrated common immune-reactive bands in all antigen types at 52 and 41 kDa against positive human and camel sera.

**Conclusion:**

This immunological study introduces camel hydatid cyst Psc as a potent diagnostic antigen and new immune-reactive fractions of 52 and 41 kDa for diagnosing hydatidosis in humans and camels.

## Introduction

Cystic echinococcosis (CE) is still one of the major zoonotic parasitic infections generated by the canine cestode parasite larval stage (hydatid cyst), *Echinococcus granulosus*, which affects humans and most domestic and feral mammals [1]. Hydatidosis is widespread, occurring in the Middle East, Egypt [2], the U.S., Australia, China, the Mediterranean, Europe, and Africa [3]. This disease has a social and economic impact on humans and livestock, causing serious losses from low productivity and organ failure in the infected animal species and human health risks associated with the cost of treating hydatid cystic disease [4]. The lifecycle of an *E. granulosus* requires two mammalian hosts: The ultimate carnivorous host (dogs or other mammals) in which the adult worm lives in the small intestine, and intermediate hosts (human and livestock, i.e., camel, cattle, and sheep), which can harbor the larval stages (metacestodes) and become advanced hydatid cysts [5]. The original host releases *E. granulosus* eggs through feces into the environment, where the intermediate hosts are infected by accidental ingestion of contaminated food or water. The oncospheres migrate through the bloodstream to different tissues and organs (particularly the liver and lungs), where they develop into hydatid cysts [6]. Within hydatid cysts, numerous tiny protoscoleces most often develop into fertile hydatid cysts by asexual multiplication that frequently develops in the liver and lungs; though they may develop in other organs, such as the kidneys, spleen, and brain [7]. In humans, hydatidosis is initially asymptomatic until the cyst becomes enlarged, causing localized pressure on internal organs and tissues. In livestock, the disease does not produce any clinical signs and is ­usually detected only during slaughterhouse inspection. Rupture of hydatid cysts often leads to sudden death from anaphylaxis, bleeding, and metastasis [8].

CE diagnosis remains difficult, although effective serological diagnoses provide opportunities for early treatment, post-treatment follow-up, and more effective chemotherapy [9]. Enzyme-linked immunosorbent assay (ELISA) is beneficial for CE diagnosis in humans and domestic animals. It is commonly inexpensive, efficient, and requires fewer trained and specialized personnel [10]. At present, hydatidosis/echinococcosis diagnosis is based on a set of imaging techniques (ultrasound, X-ray, and computerized tomography) and immunodiagnostic methods [1]. Serological techniques still lack diagnostic specificity, especially in endemic areas. Assessment and purification are required to increase the sensitivity of these techniques to detect and confirm the disease in its early stages [11]. The latex agglutination test is an appropriate and applicable diagnostic method for CE, especially when followed by confirmatory ELISA [12]. Immunoblotting is reported to yield specific and sensitive hydatidosis diagnostics. Furthermore, indirect ELISA is beneficial in primarily diagnosing CE and in post-treatment follow-up by detecting anti-*E. granulosus* IgG [13]. Camel hydatid fluid, protoscoleces-crude antigens, and sheep hydatid cysts may be helpful for efficient hydatidosis diagnoses in humans [14].

*E. granulosus*, formerly regarded as a single species with high genotypic and phenotypic diversity, is now recognized as a multiple cryptic species that considerably in morphology, development, and host specificity (including human infectivity/pathogenicity) among others. [9]. Based on phenotypic characteristics and gene sequences, *E. granulosus* (*sensu lato*) is now subdivided into *E. granulosus* (*sensu stricto*) (including G1–3), *Echinococcus felidis* (the “lion strain”), *Echinococcus equinus* (the “horse strain,” genotype G4), *Echinococcus ortleppi* (the “cattle strain,” and genotype G5), and *Echinococcus canadensis*. *E. canadensis* show the most diversity and are composed of the “camel strain” (genotype G6), the “pig strain” (genotype G7), and two “cervid strains” (genotypes G8 and G10) [7]. This nomenclatural subdivision of the agents of CE identification resulted from uniting closely related strains and genotypes after evaluating the biological and molecular tools of individual species [2]. However, several taxonomic issues are still unresolved and require additional data. At present, no reliable method is completely effective in diagnosing hydatidosis in humans and animals.

Therefore, this study aimed to prepare and purify different hydatid cyst antigens (cyst fluid, protoscoleces, and germinal layers) and to evaluate their diagnostic efficacy for detection in humans and camels using serological and immunological techniques.

## Materials and Methods

### Ethical approval

All experimental procedures were performed in accordance with the institutional guidelines of the National Research Centre’s Animal Research Committee under Ethical protocol 18/234.

### Study period and location

The study was conducted from November 2019 to June 2020. Cairo Governorate, Egypt, was selected for this study, blood samples and lung hydatid cysts were obtained from camels slaughtered in Cairo slaughterhouses, and human blood samples were collected from outpatient clinic of the Faculty of Medicine Hospital, Cairo University.

### Antigens preparation

#### Camel hydatid cyst antigens

Hydatid cyst fluid antigen (FLc)

FLc was aseptically aspirated from a hydatid cyst derived from the lung cysts of a camel, as described by Toaleb *et al*. [15].

Protoscoleces antigen (Psc)

An aseptic puncture of fertile camel hydatid cysts was adopted to obtain the protoscoleces, as described by Carmena *et al*. [16].

Hydatid cyst germinal antigen (GLc)

Cyst fluid was removed from the cysts before the cyst walls were cut, and the fluid was kept in phosphate-buffered saline (PBS, pH 7.4). GLc (inner layer) was then carefully scraped from the outer layer with forceps. The separated GLc extracts were microscopically examined to confirm the absence of traces of the outer layer of the protoscoleces [17].

#### Human hydatid cyst antigens

Hydatid cyst fluid (FLh)

FLh was prepared from aspirated fertile hepatic hydatid cyst obtained from human samples through a sterile puncture during surgery and was processed according to Gadea *et al*. [18].

Protoscoleces antigen (Psh)

Aspirated human hydatid cyst fluid was examined under a microscope for the presence of protoscoleces. Protoscolex sediment was sonicated in PBS and centrifuged for 30 min at 6000 x*g*. The supernatant was the protoscoleces’ crude antigen [16].

### Collection and preparation of sera

#### Camel sera

A total of 170 camel blood samples were obtained during slaughter in Cairo slaughterhouses. The postmortem information of these animals documented 41 positive samples (camels with lung cysts) and 23 negative samples (healthy camels free from cysts), as verified by a veterinary inspection. Afterward, 106 random camel serum samples were collected.

#### Human sera

A total of 169 blood samples were collected from an outpatient clinic in the Kasr Alainy Faculty of Medicine Hospital and from patients who complained about digestive disturbances, fever, and abdominal pain. Specifically, 26 blood samples were obtained from patients with sonographic detection of chest or liver hydatidosis or both (positive control), 17 blood samples were taken from people with no sonographic hydatidosis detection (negative control), and 126 blood samples were randomly obtained.

#### ELISA

The most potent antigen was used to diagnose hydatidosis via ELISA in random camel and human blood samples. ELISA was adopted, according to Toaleb *et al*. [19]. Antigen, sera, and conjugate concentrations were determined using a checkerboard titration, and the cutoff value was calculated by mean values of negative sera OD values + 3 standard deviation (SD)based on Elfadaly *et al*.’s method [20].

#### Antigens characterization

A 10% SDS-PAGE was performed for antigen characterization in polyacrylamide gels under reduction. The prepared antigens were individually mixed with a 2-mercaptoethanol containing buffer before loading into the gel. Fixed gel in 50% methanol was stained using a silver stain according to the method described by Wray *et al*. [21].

#### Immunoblot

All prepared antigens were electrophoresed 2 times against a prestained protein ladder on two gels and then blotted onto two nitrocellulose membranes, according to Towbin *et al***.** [22]. After washing and blotting, the nitrocellulose membrane was kept incubated with naturally infected camel sera at a 1:100 dilution. A protein-A horseradish peroxidase conjugate diluted at 1:2000 was used for immunoblotting. The second membrane was incubated with naturally infected human sera at a 1:100 dilution. Anti-human IgG horseradish peroxidase conjugate was used at a 1:1000 dilution. A 4-chloro-1-naphthol sigma solution was used to reveal the bands.

### Statistical analysis

Statistical analysis was performed using the unpaired students’ test using computer software to compare groups, sensitivity, and specificity of Psc, according to Parikh *et al*. [23]. The data were presented as mean±standard deviation.

## Results

### The potency of bound antigens with CE antibodies

**Figure-1:**
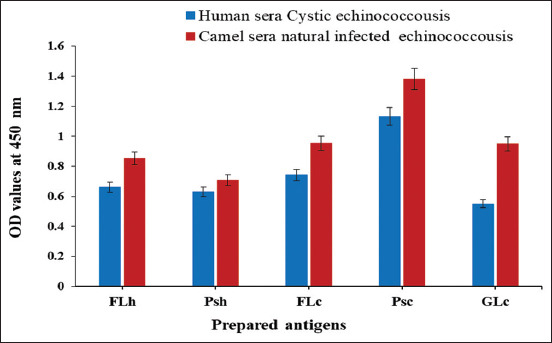
Reactive binding activities in naturally infected camel and human sera with cystic echinococcosis toward human and camel hydatid cyst antigens; FLh, Psh, FLc, Psc, and GLc.

ELISA was adopted to prove CE antibody binding activities in naturally infected human and camel sera to FLh, Psh, FLc, Psc, and GLc. The profiles bound in human and camel sera to FLc and GLc are higher than those bound to FLh and Psh. Psc to camel and human sera binding proved to be the most potent of all antigens (Figure-1).

### Sensitivity and specificity of Psc antigen

**Figure-2:**
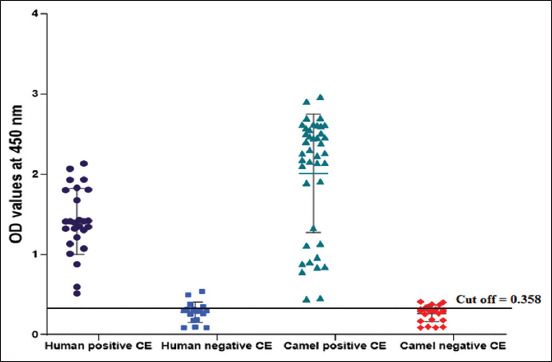
Sensitivity and specificity of Psc antigen for cystic echinococcosis in naturally infected and negative human and camel sera.

ELISA was adopted to evaluate the potency of Psc in CE diagnosis among positive and healthy camel and human sera. All positive camel and human sera reacted positively with the Psc antigen and exhibited 100% sensitivity. The specificity of Psc was recorded at 78.26% and 76.47% with healthy (negative) camel and human sera, respectively, at a cutoff value of 0.358 (Figure-2).

### Antibody IgG detection of CE in random camel and human sera

**Figure-3:**
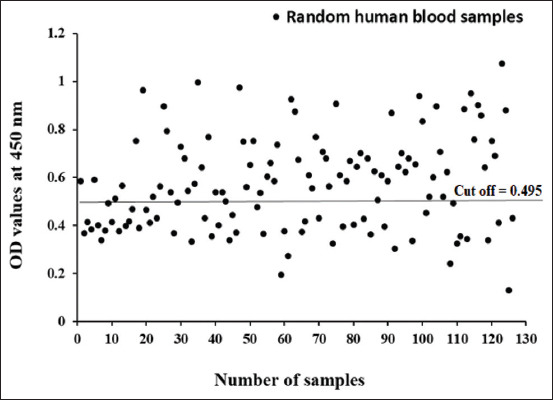
Diagnostic potency of Psc antigen in diagnosis of cystic echinococcosis in collected random human blood sera.

**Figure-4:**
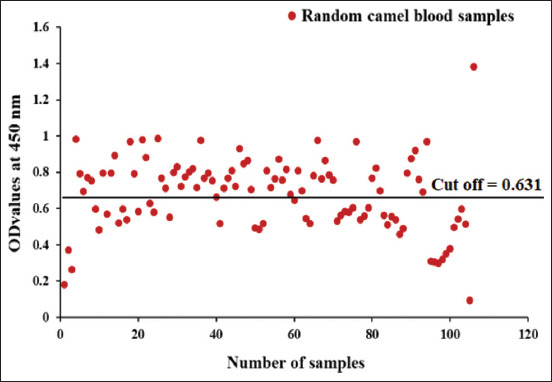
Diagnostic potency of Psc antigen in diagnosis of cystic echinococcosis in collected random camel sera.

CE antibody detection in random camel and human samples was performed using ELISA using Psc. Psc potency detected 54.79% CE antibodies in the collected random human sera at a cutoff value of 0.495 (Figure-3), whereas 61.32% of CE antibodies were detected in random camel sera at a cutoff value of 0.631 (Figure-4).

### Electrophoretic profile of the five antigens

**Figure-5:**
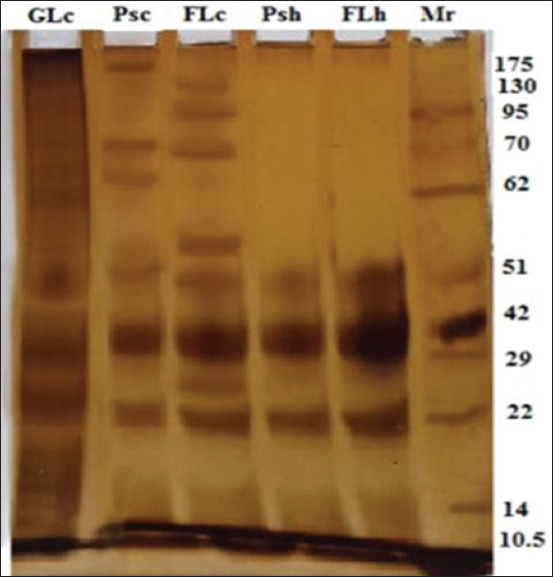
Comparative SDS-PAGE pattern of five antigens; three camel hydatid cyst antigens; GLc, Psc, and FLc, and two human hydatid cyst antigens Psh and FLh, and molecular weight standards in kDa; lane Mr.

Electrophoresis of the five antigens; FLh, Psh, FLc, Psc, and GLc at 10% SDS-PAGE under reduction demonstrated extensive electrophoretic similarity, especially in three bands (52, 41, and 22 kDa). Nevertheless, exclusive molecules to GLc were detected at 100, 71, 68, 60, 45, 20, 17, and 10 kDa. Psc was also detected at 205, 71, and 68 kDa, whereas, in FLc, six bands were detected at 200, 135, 95, 71, 58, 45, and 14 kDa (Figure-5).

### Immunogenic reactive components of antigens by immunoblotting

**Figure-6:**
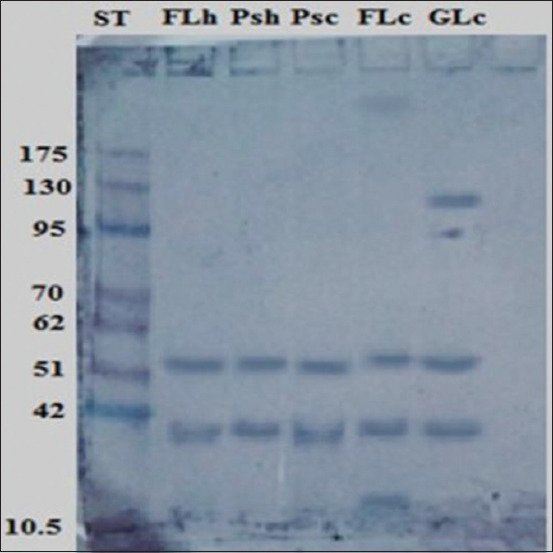
Identification of immunogenic bands recognized by naturally infected camel sera in FLc, Psc, GLc, FLh, and Psh antigens. Molecular weight standards in kDa are demonstrated in lane St.

**Figure-7:**
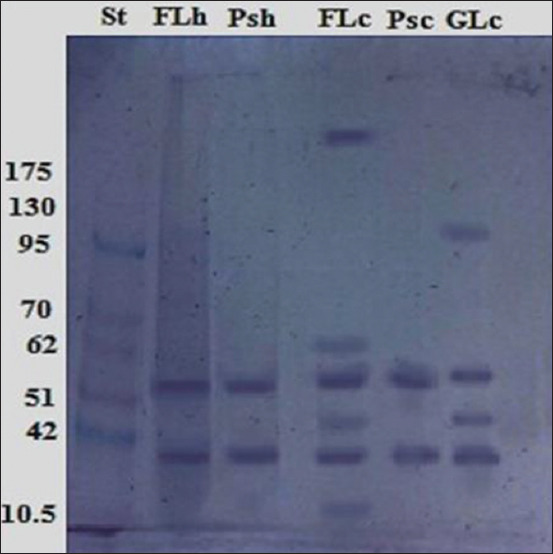
Identification of immunogenic bands recognized by naturally infected human sera in FLc, Psc, GLc, FLh, and Psh antigens. Molecular weight standards in kDa are demonstrated in lane St.

Immunogenic reactive components recognized by naturally infected camel sera shared two common bands at the same molecular weights (52 and 41 kDa). Furthermore, 200 and 22 kDa were recognized in FLc and 100 kDa in GLc, as proven by immunoblotting (Figure-6). Furthermore, naturally infected human sera identified the same five common immunogenic bands at 52 and 41 kDa. Other immunogenic bands at 200, 58, 45, and 14 kDa were detected in FLc and 100 and 45 kDa were detected in GLc (Figure-7).

## Discussion

CE is a major constraint on both public health and the economy. This disease was recently included in the WHO strategic plan to combat neglected tropical ­diseases [24]. In livestock, CE is asymptomatic and is usually diagnosed in necropsy. Serological and immunological assays have been found to be beneficial in initial human and animal diagnoses, improvements in the quality of disease management, and following human surgery or drug treatment [25]. This study investigated the potency of five hydatid cyst antigens (FLh, Psh, FLc, Psc, and GLc) to detect the binding activities of CE antibodies in naturally infected camel and human sera using ELISA. Psc proved to be the most potent antigen. FLc and GLc ranked higher than FLh and Psh in camel and human sera-binding capabilities. These results follow those previously reported by Pagnozzi *et al*. [4] who also surveyed camel and human CE cases using ELISA; they demonstrated that the protoscolex was the most efficient diagnostic antigen.

ELISA is represented as the choice test for CE diagnosis in humans and domestic animals [26]. It is generally inexpensive, efficient, of good sensitivity and specificity, unsophisticated, and requires fewer trained personnel [27]. In this work, Psc revealed 100% sensitivity and 78.26% and 76.47% specificity to serologically tested camel and human sera, respectively, using ELISA. Our results agree with those reported by Simsek and Koroglu [13], who found that protoscoleces sensitivity and specificity were 100% and 75%, respectively, using ELISA. The CE prevalence adopted by ELISA in this study was 54.79% and 61.32%, in randomly collected camel and human serum samples, respectively. Because of recent CE control programs, the prevalence of CE infection has decreased significantly. However, CE is still a common health problem in developing countries, particularly in rural areas [28]. Community-based studies have demonstrated that the prevalence of human CE is between 1% and 10% in endemic regions [14]. Notably, 22.6% of slaughtered camels came from the Northern and Southern Egyptian slaughterhouses [29]. The discrepancy in the prevalence of CE in human and slaughtered camels was due to the type of serological tests used, the effectiveness and purification of the antigens used, and seasonal factors [30].

The use of crude hydatid cyst antigens in serological diagnostic tests is inadequate. Thus, the purification of hydatid cyst antigens excludes other cross-reactive proteins [14]. Many types of electrophoretic analysis have been used to purify hydatid antigens. In this study, 10% SDS-PAGE under reduction demonstrated extensive electrophoretic similarity between the five purified hydatid cyst antigens (FLh, Psh, FLc, Psc, and GLc), especially in three common shared reactive bands (52, 41, and 22 kDa). This method verifies a previous yield of a most prominent paramyosin antigen fraction of hydatid cyst fluid by SDS-PAGE analysis (65, 45, and 29 kDa) [26]. It also identified results of shared antigenic components of hydatid cyst fluid antigen and protoscoleces at 20, 54, and 65 kDa. Unfortunately, it is without detection of the diagnostic potentials of these purified antigenic components [16].

Western immunoblotting of different antigens, including HC fluid, protoscoleces, and cyst wall antigens, has been useful for hydatid cyst diagnoses [31]. In this research study, immunoblotting demonstrated that common immune-reactive bands in all purified hydatid cyst antigen types at 52 and 41 kDa against naturally infected human and camel sera besides other immunogenic bands of 200 and 100 kDa recognized in FLc and GLc antigens, respectively. In the same way, Nazari *et al*. [32] reported that infected hydatid cyst sera can react with specific bands of crude antigens prepared from the hydatid cyst fluid, protoscolex, and cyst walls. They also concluded that a 20-kDa band of sheep hydatid cyst fluid is an efficient serodiagnostic antigen for human hydatidosis. Another study proves that hydatid cyst fluid immunoblotting exhibits 60, 38, and 22 kDa antigenic bands when examined with naturally infected camel sera [11].

## Conclusion

In this investigation, diagnostic antigens prepared from camel hydatid cysts can be successfully used to diagnose hydatidosis in both camel and human sera using the ELISA test, which is useful to control infection and reduce human transmission. Moreover, this sero-immunological study introduces camel hydatid cyst protoscoleces as potent diagnostic antigens and new immune-reactive fractions of 52 and 41 kDa for the diagnosis of human and camel CE.

## Authors’ Contributions

MAH, NIT and RMS conceived and designed the experiment. NIT collected blood samples and lung hydatid cysts from slaughtered camels. NAH, AMMH, and ABY collected and aspirated human hydatid cyst fluid and prepared antigens. NIT and RMS carried out SDS-PAGE under reducing conditions, western blot, and ELISA for detection of CE specific antibodies. NIT and RMS analyzed the data, represented it in figures and wrote the paper. All authors revised and approved the final manuscript.
